# Interpersonal Family Dynamics Relate to Hippocampal CA Subfield Structure

**DOI:** 10.3389/fnins.2022.872101

**Published:** 2022-06-17

**Authors:** Christine Coughlin, Eliya Ben-Asher, Hannah E. Roome, Nicole L. Varga, Michelle M. Moreau, Lauren L. Schneider, Alison R. Preston

**Affiliations:** ^1^Center for Learning and Memory, The University of Texas at Austin, Austin, TX, United States; ^2^Department of Psychology, The University of Texas at Austin, Austin, TX, United States; ^3^Department of Neuroscience, The University of Texas at Austin, Austin, TX, United States

**Keywords:** social environment, social relationships, family interaction, development, medial temporal lobe

## Abstract

Social environments that are extremely enriched or adverse can influence hippocampal volume. Though most individuals experience social environments that fall somewhere in between these extremes, substantially less is known about the influence of normative variation in social environments on hippocampal structure. Here, we examined whether hippocampal volume tracks normative variation in interpersonal family dynamics in 7- to 12-year-olds and adults recruited from the general population. We focused on interpersonal family dynamics as a prominent feature of one’s social world. Given evidence that CA_1_ and CA_2_ play a key role in tracking social information, we related individual hippocampal subfield volumes to interpersonal family dynamics. More positive perceptions of interpersonal family dynamics were associated with greater CA_1_ and CA_2/3_ volume regardless of age and controlling for socioeconomic status. These data suggest that CA subfields are sensitive to normative variation in social environments and identify interpersonal family dynamics as an impactful environmental feature.

## Introduction

Our social interactions influence our thoughts and feelings on a daily basis. An argument with a parent might dampen our day, while a kind gesture from a sibling could enhance it. While research indicates extreme variation in social interactions influences children’s neural development (Teicher and Samson, 2016; VanTieghem et al., 2021), much less is known about the impact of normative variation (Belsky and de Haan, 2011). Here, we examine whether normative variation in interpersonal family dynamics tracks hippocampal volume in 7- to 12-year-olds and adults. These dynamics encompass how individual family members relate to one another, including their feelings and patterns of behavior. We reasoned that hippocampal structure may be associated with this ubiquitous feature of our social world given its sensitivity to features of one’s social environment (Paylor et al., 1992; Olson et al., 2006; Teicher and Samson, 2016; VanTieghem et al., 2021), as well as its demonstrated role in social cognition (Hitti and Siegelbaum, 2014; Alexander et al., 2016; Smith et al., 2016).

A large portion of work examining the relation between variation in social experience and hippocampal volume in children has focused on extreme adversity. Importantly, this extreme adversity has often been grounded in factors related to family. Early institutional care (VanTieghem et al., 2021), childhood maltreatment (Riem et al., 2015), negative life events (Gerritsen et al., 2015), and neighborhood poverty (Taylor et al., 2020)—factors which are all part of the broader family context—have all been associated with smaller hippocampal volume. Negative parenting itself (e.g., maternal hostility, intrusiveness, and negative affect) has a detrimental impact on hippocampal structure, leading to smaller volumes, which is mediated through the cortisol pathway (Blankenship et al., 2019). Cortisol release as part of the stress response reduces hippocampal plasticity and leads to neurotoxicity in extreme cases (Foy et al., 1987; Kim and Yoon, 1998; Kim et al., 2015; McEwen et al., 2016), changes which would be reflected in smaller hippocampal volumes. While normative variation in interpersonal family dynamics differs from extreme adversity, negative family interactions may elicit a cortisol response, which may impact hippocampal structure if such negative interactions are habitual or chronic (Blankenship et al., 2019).

Although most prior studies have focused on the relation between negative family environments and hippocampal volume, a handful of longitudinal studies have further investigated how hippocampal volume varies with positive parental care. One study found that maternal support during early childhood predicted larger hippocampal volume at school age (Luby et al., 2012), while others found a non-existent (Whittle et al., 2014) or negative (Rao et al., 2010) relationship between maternal support and hippocampal structure. Though findings are mixed and were obtained across diverse samples (e.g., children who were exposed to cocaine *in utero* in Rao et al., 2010), together they suggest that factors related to family function may sometimes exert a positive impact on human hippocampal structure. This possibility is supported by a more extensive body of work that has investigated positive social influences on hippocampal structure using rodent models.

While rodent models do not share a family structure with humans, they have provided useful insight in areas for which human data are not readily available, including impacts on hippocampal structure and function at the molecular, cellular, and circuit levels. Research that involves an extreme manipulation of one’s environment, such as enriched environment paradigms, is one such area. Enriched environment paradigms involve a combination of social and inanimate stimulation (Rosenzweig et al., 1978; Rosenzweig and Bennett, 1996), with rodents often housed in larger groups and cages with more toys and nesting materials (Kempermann, 2019). Placing rodents in these environments leads to a greater number of hippocampal synaptic contacts (Kempermann, 2008) and neurons (Kempermann et al., 1997), along with larger overall hippocampal volume (Hüttenrauch et al., 2016). These benefits may partly arise from increased opportunities for social interaction that ultimately result in a more developed social life (Zarif et al., 2018; Kempermann, 2019). It is possible that positive interpersonal family dynamics support a similar opportunity for increased social interaction in humans, potentially leading to enhanced hippocampal volume.

Rodent models have also provided insight into how the cellular properties of individual hippocampal subfields may be sensitive to social experience. Work with these models has found that the hippocampus contains receptors for oxytocin and vasopressin (Schafer and Schiller, 2018), two neuropeptides that mediate social behaviors (Insel, 2010). Increases in these neuropeptides during social encounters may modulate hippocampal plasticity, especially in the CA_2_ subfield for which receptor concentrations are particularly high (Cilz et al., 2019). Furthermore, direct activation of vasopressin receptors within CA_2_ improves memory for social experiences (Smith et al., 2016). Enhanced CA_2_ modulatory effects can also extend to CA_1_, with oxytocin increasing the excitability of CA_1_-CA_2_ transmission in rodents (Pagani et al., 2015; Lin et al., 2018). Together, this rodent work indicates that the hippocampus—particularly the CA_1_ and CA_2_ subfields—may exhibit cellular sensitivity to a wide range of social experiences and information. Extant human work complements this possibility by highlighting a critical role of the hippocampus in social cognition and memory (Laurita and Spreng, 2017). The hippocampus not only helps humans remember social relations (Benoy et al., 2018), but also tracks features important for understanding their social environment including information on social hierarchy (Kumaran et al., 2012), rank order (Kumaran et al., 2016), and affiliation (Tavares et al., 2015). Here, we bring together these rodent and human lines of work to test whether human subfields CA_1_ and CA_2_ are particularly sensitive to social interactions within one’s family.

We build upon prior work by investigating social experience along a continuum, testing whether hippocampal structure tracks normative variation in interpersonal family dynamics within a developmental sample. We examined this question in 7- to 12-year-olds and adults because the hippocampus may be more sensitive to the environment earlier in life (Casey et al., 2000; Andersen et al., 2008; Tottenham and Sheridan, 2010; Humphreys et al., 2019). Participants were asked to report their perceptions of their current interpersonal family dynamics (i.e., how they viewed interactions within their present-day family, at their current age). These perceptions were then related to individual hippocampal subfield volumes (CA_1_, CA_2/3_, dentate gyrus, and subiculum) given prior evidence that hippocampal subfields may be differentially sensitive to environmental features (Teicher et al., 2012; Tzakis and Holahan, 2019). We tested the hypothesis that more positive interpersonal family dynamics would relate to greater hippocampal volume, particularly within the CA_1_ and CA_2_ subfields given their sensitivity to social stimuli (Pagani et al., 2015; Lin et al., 2018; Cilz et al., 2019). We also hypothesized that the relationship between family dynamics and hippocampal volume may be greater earlier in life, when continued development of the hippocampus may enhance its structural plasticity (Casey et al., 2000; Andersen et al., 2008; Tottenham and Sheridan, 2010; Humphreys et al., 2019).

## Materials and Methods

### Design

The data presented in this paper were collected as part of a project examining the relation between one’s environment and hippocampal structure during development. Participants were enrolled in this project if they participated in any subsidiary developmental neuroimaging study within the lab. Though these subsidiary studies focused on diverse aspects of cognition, each involved collection of high-resolution structural MR data using an identical acquisition protocol, as well as a validated assessment of interpersonal family dynamics. These commonalities allowed us to assess the relationship between hippocampal volume and interpersonal family dynamics within a large cross-sectional sample.

### Participants

The final sample included 149 participants of whom 91 were children (7–12 years; *M* = 9.75; SD = 1.57; 50 female) and 58 were adults (18–33 years; *M* = 23.90; SD = 4.26; 28 female) (see Supplementary Material for detailed information about screening measures and participant exclusions). Two participants included in the final sample were missing data for only one of the relevant variables [i.e., socioeconomic status (SES) or sex]. Because all analyses included one of these variables (but never both together), one participant was omitted from each analysis (adjusted *N* = 148). All participants were recruited from the Austin-area community in Texas. Informed consent (if adult participant) or parental consent and child assent (if child participant) were obtained prior to participation. Participants received monetary compensation and possibly a small prize in appreciation of their time. This study was approved by the Institutional Review Board at The University of Texas at Austin.

### Materials

#### The Systemic Clinical Outcome and Routine Evaluation-15

Participants’ perceptions of interpersonal family dynamics were assessed using the Systemic Clinical Outcome and Routine Evaluation-15 (SCORE-15; if 12-years-old or older; Stratton et al., 2010) and the child SCORE-15 (if younger than 12-years-old; Jewell et al., 2013). Both instruments have been established as reliable and valid self-report measures of family function (Carr and Stratton, 2017). When completing the SCORE-15, participants are asked to report on how they view their family currently (in the present, at their current age, regardless of whether they are a child or adult). Those administered the original version of the SCORE-15 (participants ages 12 years and older) are told that “family” is often used to describe the people with whom one lives, but that this need not be the case. They are also instructed to choose whom they want to count as their family (thus “family” does not have to reflect the people with whom they live and/or share a biological relationship). Participants then rate 15 statements according to how well they describe their family on a five- or six-point Likert scale ranging from not at all (1) to very or extremely well (5 or 6, depending on the version). The original adult version uses a “6” as the upper end of the scale. The child version uses a “5” as the upper end because it was found that negligible information is lost when the scale is reduced by one point (Carr and Stratton, 2017).

The same 15 statements are included in the adult and child versions, though the wording is slightly simplified in the latter. Together, these statements provide an overall measure of interpersonal family dynamics. However, statements can also be organized into three factors that each reflect a unique dimension of family function: a *strength and adaptability* dimension that reflects the degree of positive communication or coping skills within the family (e.g., “We are good at finding new ways of dealing with things that are difficult”); an *overwhelmed by difficulties* dimension that reflects the perceived difficulty of handling hardships within the family (e.g., “We find it hard to deal with everyday problems”; and, a *disrupted communication dimension* that reflects unhealthy communication or negative interpersonal dynamics within the family (e.g., “People don’t often tell each other the truth in my family”).

##### Systemic Clinical Outcome and Routine Evaluation-15 Scoring

The SCORE-15 was scored so that higher overall scores would reflect more positive perceptions of interpersonal family dynamics. First, negatively worded items (e.g., “We find it hard to deal with everyday problems”) were reverse-scored. Next, the sum of each participant’s responses across all 15 items was computed. This sum was then divided by the highest possible sum the participant could have received given the version of the SCORE-15 they were administered (i.e., the child or adult version, depending on their age). This approach put overall scores on a percentage scale that was equivalent across child and adult participants. Given our interest in global interpersonal family dynamics, overall scores were of primary interest. However, scores within each dimension (computed taking the same approach) were used in exploratory analyses.

#### Socioeconomic Status Assessment

A parental-report (if child participant) or self-report (if adult participant) questionnaire was used to obtain the highest level of educational attainment achieved by the participant’s parents (adapted from Engelhardt et al., 2019). Parent educational attainment for those with parents ages 25+ years serves as a good univariate proxy for SES (American Psychological Association Task Force on Socioeconomic Status, 2002). Annual income was not considered because it does not reflect accumulated wealth and individuals are often reluctant to report it (Oakes and Rossi, 2003).

##### Socioeconomic Status Scoring

Each parent’s educational attainment was scored on a 4-point Likert scale where “1” = no high-school diploma, “2” = high school diploma, “3” = bachelor’s degree, and “4” = graduate degree. The sum of both parent’s educational attainment was computed to yield the participant’s SES score.

### Procedure

#### Session 1

In an initial behavioral session, participants completed exposure to a practice MRI environment, screening measures (ensuring IQ and psychiatric symptoms within the normal range; see Supplementary Material), cognitive tasks unrelated to the present study, and the SES and SCORE-15 (Stratton et al., 2010; Jewell et al., 2013) questionnaires. The SCORE-15 was verbally administered to participants ages nine and younger to ensure comprehension; all other participants completed it independently with an experimenter nearby to answer questions if needed.

#### Session 2

Participants returned to the lab approximately one month later (*M* = 1.20 months; SD = 1.24) for a second session during which they completed cognitive tasks (unrelated to the present study) while MRI scans were collected. Across all subsidiary studies, we used an identical acquisition sequence to obtain high-resolution structural MRI scans for hippocampal volume estimation (see section “MR Data Acquisition” for details). Several steps were taken to reduce participant motion and anxiety during these scans (e.g., participants were allowed to watch a child-friendly movie, were covered with a weighted blanket, and had padding placed between their head and the head coil).

### MR Data Acquisition

Imaging data were collected on a 3T Siemens Skyra MRI at The University of Texas at Austin Biomedical Imaging Center. One to two (or three, if one of the first two images was of poor quality and timing allowed) high-resolution coronal T2-weighted structural scans were collected perpendicular to the hippocampal long-axis (TR = 13,150 ms, TE = 82 ms, 0.4 mm × 0.4 mm in-plane resolution, 1.5 mm thru-plane resolution, 60 slices). When two coronal images of acceptable quality were acquired (determined by visually inspecting images for artifacts or positioning that prevented visualization of the hippocampal structure) for a single participant, images were co-registered using ANTS (Avants et al., 2011) and averaged to boost the signal-to-noise ratio (SNR), creating a single mean coronal image. A whole-brain T1-weighted 3-D MPRAGE volume (TR = 1,900 ms, TE = 2.43 ms, flip angle = 9°, 1 mm isotropic voxels) was also collected to estimate overall intracranial volume.

### Hippocampal Subfield Definition

Hippocampal subfield regions of interest (ROI) were defined using the Automated Segmentation of Hippocampal Subfields (ASHS) software (version 0.1.0, rev 103; Yushkevich et al., 2015). Specifically, we used ASHS with a custom developmental atlas by Schlichting et al. (2019) to extract volumes for hippocampal CA_1_, CA_2/3_, DG, and subiculum subfields in each participant’s native space (see Figure 2A for example hippocampal ROIs). Consistent with other approaches (e.g., Dalton et al., 2017; Ho et al., 2017), this atlas groups CA_2_ and CA_3_ together given difficulty distinguishing between these subfields on MRI scans of this resolution. Extracted volumes for each individual subfield encompassed the entire longitudinal axis of the hippocampus with the exception of its most posterior slices for which reliable subfield delineation was not possible during atlas creation (see Schlichting et al., 2019 for details). Extracted hippocampal subfield volumes were visually inspected to ensure mislabeling had not occurred. No subjects needed to be excluded based on this visual inspection. No manual editing of extracted volumes was performed.

**FIGURE 1:**
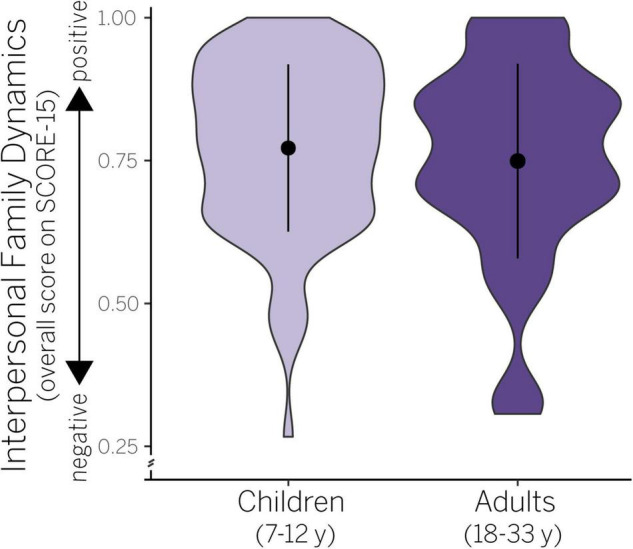
Violin plots showing probability density (width of shaded data) and mean/SD (black circle, vertical bar) in interpersonal family dynamics in children and adults. Raw scores are plotted ranging from 0 (negative) to 1 (positive).

**FIGURE 2:**
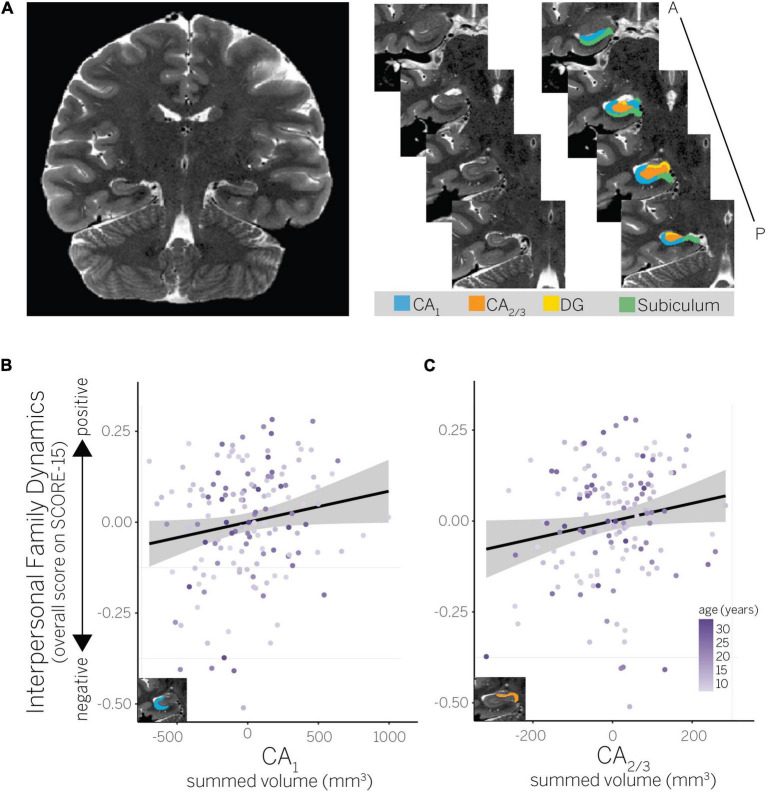
**(A)** Example hippocampal subfield ROIs (CA_1_, CA_2/3_, dentate gyrus, and subiculum) for a representative child participant. ROIs are shown on the right hemisphere, but were summed bilaterally for all analyses. **(B)** Partial residual plot showing association between perceived interpersonal family dynamics and CA_1_ volume, and **(C)** interpersonal family dynamics and CA_2/3_ volume.

### Intracranial Volume Adjustment

We estimated intracranial volume (ICV) from each participant’s T1-weighted structural image using Freesurfer (Desikan et al., 2006). For each ROI, we conducted a regression of raw volume on ICV, age group, and ICV × age group. Because the ICV × age group interaction was not significant for any ROI (*p*s > 0.15), children and adults were combined for all ICV adjustments. Extracted individual hippocampal subfield volumes were adjusted for differences in head size using an approach similar to prior work (Raz et al., 2005; Schlichting et al., 2017, 2019). Resulting ICV-adjusted volumes were used in all analyses.

### Statistical Analyses

Analyses were done using R (version 3.6.3; R Core Team, 2020) with the lme4 (version 1.1.26; Bates et al., 2015), stats (version 3.6.3; R Core Team, 2020), and car (version 3.0.10; Fox and Weisberg, 2019) packages. The reports (version 0.5.0; Makowski et al., 2020) and gplot2 (version 3.3.3; Wickham, 2016) packages were also used to support reproducibility in results reporting and data visualization. For all analyses, age was measured in months, SES was measured as the sum of parents’ educational attainment, interpersonal family dynamics was measured as overall score on the SCORE-15 (or on a specific SCORE-15 dimension when specified), and individual subfield volumes were measured by combining volume across both hemispheres for each subfield, respectively (bilateral CA_1_, CA_2/3_, DG, subiculum, and posterior hippocampus). SES and sex data were missing for one participant each. Because all analyses included one of these variables (but never both together), the final sample was reduced by one for each analysis. See Table 1 for descriptive statistics for screening, demographic, and questionnaire measures.

**TABLE 1:** Sample descriptives.

Measures	Entire sample (*n* = 149)		Children (*n* = 91)		Adults (*N* = 58)	
	Range	*M* (SD)	Range	*M* (SD)	Range	*M* (SD)
**Screening measures**						
CBCL (0–226)	–	–	0–79	19.48 (13.26)	–	–
SCL (0–4)	–	–	–	–	0.00–1.43	0.24 (0.23)
WASI FSIQ-2	94–193	119.69 (12.62)	94–145	120.81 (10.67)	94–193	117.98 (15.08)
**Main measures**						
Age (years)	7.08–33.40	15.38 (7.55)	7.08–12.80	9.75 (1.57)	18.90–33.40	23.90 (4.26)
SCORE-15 (0–1)	0.27–1.00	0.76 (0.15)	0.27–1.00	0.77 (0.15)	0.31–1.00	0.75 (0.17)
SES (1–8)	3–8	6.32 (1.29)	3–8	6.40 (1.15)	4–8	6.20 (1.49)

*M and SD are used to represent mean and standard deviation.*

#### Preliminary Analysis of Variability in Interpersonal Family Dynamics

A preliminary analysis examined whether interpersonal family dynamics varied by age and sex. Two nested linear models were run (using stats:lm) and then compared (using stats:anova; Table 2). The first was a *main effects model* which included only main effects of age and sex; the second was an *interaction model* which added an interaction between these variables. An *F*-test comparing the two models found that adding an interaction term did not result in a significantly improved fit, *F*(1,144) = 0.26, *p* = 0.608. Statistics for the *main effects model*, the best fitting model, are thus reported. *F*-tests were used to assess the significance of each predictor included in this model (using car:Anova, type II sum of squares).

**TABLE 2:** Models compared for main analyses.

Models	Model comparison tests
**Models of interpersonal family dynamics**	
(1) Main effects model: FamDyn ∼ Age + Sex	–
(2) Interaction model: FamDyn ∼ Age + Sex + Age:Sex	Model 1 vs. 2: *F*(1,144) = 0.26, *p* = 0.608
**Models of hippocampal subfield volumes**	
(1) Main effects model: SubVol ∼ Age + SubID + FamDyn + SES + (1| Pt)	–
(2) Subfield interaction model: SubVol ∼ Age + SubID + FamDyn + SES + FamDyn:SubID + (1| Pt)	Model 1 vs. 2: χ^2^(4) = 9.62, *p* = 0.047 *
(3) 3-way interaction model: SubVol ∼ Age + SubID + FamDyn + SES + FamDyn:SubID + FamDyn:Age + SubID:Age + FamDyn:SubID:Age + (1| Pt)	Model 2 vs. 3: χ^2^(9) = 11.89, *p* = 0.220

Family dynamics (FamDyn); participant (Pt); subfield ID (SubID); subfield volume (SubVol). *p < 0.05.

#### Interpersonal Family Dynamics and Hippocampal Subfield Volumes

Our central analyses tested the hypothesis that positive interpersonal family dynamics would be associated with larger hippocampal subfield volumes. Given extant rodent data, we predicted that the relationship between these factors would vary across individual hippocampal subfields, wherein significant relationships may be most apparent in CA_1_ and CA_2/3_ (Pagani et al., 2015; Lin et al., 2018; Cilz et al., 2019). Our analyses therefore tested for an interaction between individual hippocampal subfield volumes and interpersonal family dynamics (see description of *subfield interaction model* below). We also considered the possibility that the association between interpersonal family dynamics and hippocampal subfield volumes may be greater earlier in development, when continued development of the hippocampus may lead to enhanced structural plasticity (Casey et al., 2000; Andersen et al., 2008; Tottenham and Sheridan, 2010; Humphreys et al., 2019). We thus tested an interaction between all three factors of interest—age, individual hippocampal subfields, and interpersonal family dynamics—in a separate model (see description of *3-way interaction model* below).

Predictions were tested using a model comparison approach. Specifically, three nested linear mixed-effects models were run (using lme4:lmer) and then compared (using stats:anova) to determine which model best fit the data (Table 2). The first model included only main effects; this model included variables for age, volumes for each bilateral hippocampal subfield (CA_1_, CA_2/3_, DG, subiculum, and posterior hippocampus), and SCORE-15. Participants thus contributed one data point each for each variable. This *main effects model* also included SES as a covariate, given its association with family processes (Conger et al., 2010) and hippocampal structure (Brito and Noble, 2014), and participant as a random factor. The remaining two models included all of the main effects, SES as a covariate, and participant as a random effect, while progressively adding interaction terms. The *subfield interaction model* added a two-way interaction between hippocampal subfield volumes and interpersonal family dynamics as measured by SCORE-15. This model allowed us to test the prediction that the association between individual hippocampal subfield volumes and interpersonal family dynamics would vary between individual subfields. The *3-way interaction model* added an additional interaction between age, individual hippocampal subfield volumes, and interpersonal family dynamics (as well as the lower-level, two-way interactions). This model allowed us to test the prediction that the association between individual hippocampal volumes and interpersonal family dynamics would vary by age.

Likelihood ratio chi-squared tests were used to determine which of these models best fit the data. The first test found that the *subfield interaction model* resulted in a significantly better fit than the *main effects model*, χ^2^(4) = 9.62, *p* = 0.047. An additional test found that the *3-way interaction model* did not result in a significantly better fit than the *subfield interaction model* [χ^2^(9) = 11.89, *p* = 0.220]. Statistics for the *subfield interaction model*, the best fitting model, are thus reported. Wald Chi-square tests were used to assess the significance of each fixed effect included in this model (using car:Anova, type III sum of squares).

#### Exploring Influence of Unique Interpersonal Family Dynamics Dimensions

Previewing results, we found that interpersonal family dynamics positively predicted volume within the CA_1_ and CA_2/3_ subfields. While our interests lie in the influence of overall interpersonal family dynamics on hippocampal subfield structure, prior studies have shown that the three dimensions of the SCORE-15 constitute unique factors of family life (Carr and Stratton, 2017). We therefore performed an exploratory *post hoc* analysis examining whether the relation between interpersonal family dynamics and these hippocampal subfield volumes appeared driven by a particular dimension (i.e., strength and adaptability, overwhelmed by difficulties, or disrupted communication). For both CA_1_ and CA_2/3_, we ran a subset of three linear models (using stats:lm; Supplementary Table 1). Each of these models included a different dimension of interpersonal family dynamics, age, and SES as predictors of individual subfield volumes (*dimension models 1–3*: CA_1_ or CA_2/3_ volume ∼ unique dimension of interpersonal family dynamics + age + SES). We then used Akaike information criterion (AIC) tests (using stats:AIC) to compare the fit of these models to models that instead included the original measure of overall interpersonal family dynamics as a predictor (*overall dynamics models:* CA_1_ or CA_2/3_ volume ∼ interpersonal family dynamics + age + SES). AIC tests were used because they allow for the comparison of non-nested models fit to the same data. The AIC statistics for these models are reported.

## Results

### Variability in Interpersonal Family Dynamics

We first assessed whether age and sex accounted for variance in interpersonal family dynamics as measured by the SCORE-15. We found that the *main effects model* best explained our data (Table 2), but was not significant, adj. *R*^2^ = −0.01, *F*(2,145) = 0.45, *p* = 0.638. According to this model, neither age [*F*(1,145) = 0.65, *p* = 0.423] nor sex [*F*(1,145) = 0.23, *p* = 0.631] predicted variance in SCORE-15 values (see also Supplementary Table 2). Thus, while there was variability in interpersonal family dynamics (Figure 1), it was not attributable to age or sex within our sample.

### Relationship Between Interpersonal Family Dynamics and Hippocampal Subfield Volumes

Our prediction was that more positive perceptions of interpersonal family dynamics would be associated with larger hippocampal subfield volumes. We further predicted this relationship may be greater for the CA_1_ and CA_2_ subfields, as well as earlier in development. We found that our data were best explained by the *subfield interaction model*, which included an interaction between individual hippocampal subfields and interpersonal family dynamics, but not interactions with age (Table 2). This model had strong explanatory power, conditional *R*^2^ = 0.96; marginal *R*^2^ = 0.94. According to this model, the relationship between hippocampal subfield volumes and interpersonal family dynamics varied by subfield [χ^2^(4) = 9.56, *p* = 0.048]. In contrast, neither age [χ^2^(1) = 2.05, *p* = 0.152] nor SES [χ^2^(1) = 0.05, *p* = 0.828] emerged as significant predictors of hippocampal subfield volumes (see also Supplementary Table 3).

To further interrogate the interaction between hippocampal subfield volumes and interpersonal family dynamics, we used linear models (using stats:lm) to quantify the extent to which interpersonal family dynamics predicted hippocampal subfield volumes within a specific ROI (CA_1_, CA_2/3_, DG, subiculum, or posterior hippocampus), while simultaneously accounting for age and SES. *F*-tests were used to assess the significance of each predictor included in these model (using car:Anova, type II sum of squares). Results showed that positive interpersonal family dynamics predicted larger hippocampal subfield volumes in CA_1_ [*F*(1,144) = 4.07, *p* = 0.045; Figure 2B] and CA_2/3_ [*F*(1,144) = 4.13, *p* = 0.044; Figure 2C], but not in any other subfield [*F*s(1,144) ≤ 0.09, *p*s ≥ 0.767]. The only other significant predictor across these models was age, which also positively predicted larger hippocampal subfield volumes in CA_2/3_ [*F*(1,144) = 20.27, *p* < 0.001; all other predictors: *F*s(1,144) ≤ 2.72, *p*s ≥ 0.101]. Although our model comparison approach did not support the inclusion of age interactions, we confirmed that the interaction between age and interpersonal family dynamics did not significantly predict hippocampal subfield volumes in any ROI, *F*s(1,143) ≤ 0.97, *p*s ≥ 0.327 (using stats:lm to run models; using car:Anova, type III sum of squares to assess the significance of each predictor). Thus, the interaction between subfield ROIs and interpersonal family dynamics was driven by positive dynamics predicting greater hippocampal volumes in CA_1_ and CA_2/3_, but not the other subfields. That age also predicted greater hippocampal volumes in CA_2/3_ suggests there may be continued developmental change in this subfield from middle childhood to adulthood (the age range targeted by our sample).

### Exploring Influence of Unique Interpersonal Family Dynamics Dimensions

A *post hoc* exploratory analysis examined whether one of the three dimensions of interpersonal family dynamics appeared to drive its relation with CA_1_ and CA_2/3_ subfield volumes. Lower AIC values were observed for models of subfield volumes that included overall family dynamics as a predictor versus those that included a unique dimension of these dynamics as a predictor, though AIC values were highly similar across models (Supplementary Table 1). Results suggest the relation between interpersonal family dynamics and both CA_1_ and CA_2/3_ subfield volumes is not driven by a particular dimension of family dynamics as measured by the SCORE-15.

## Discussion

Here, we show that CA_1_ and CA_2/3_ structure tracks normative variation in a key feature of our social environment—interpersonal family dynamics—in children and adults. In line with our predictions, we found that positive perceptions of interpersonal family dynamics were associated with greater CA_1_ and CA_2/3_ volumes. Perceptions of family dynamics mattered in general, with no clear evidence that a single dimension of family dynamics drove the association with CA_1_ and CA_2/3_ volumes. These effects were independent of age and significant even when controlling for SES, a variable known to impact hippocampal volume (Brito and Noble, 2014). While prior work has shown that hippocampal structure is sensitive to one’s environment (McEwen, 2012; Brito and Noble, 2014; Blankenship et al., 2019), the majority of this work has examined the influence of extreme adversity or enrichment (Paylor et al., 1992; Olson et al., 2006; Riem et al., 2015; Teicher and Samson, 2016; VanTieghem et al., 2021). Although informative, such findings do not easily generalize to the subset of the population for whom environmental experience is less extremely negative or positive. Our findings therefore extend prior work in both rodents and humans by demonstrating a link between normative variation in family dynamics and hippocampal structure. Furthermore, by leveraging high-resolution neuroimaging methods, we were able to show that this association is specific to the CA_1_ and CA_2/3_ subfields, extending convergent observations in rodents (Pagani et al., 2015; Smith et al., 2016; Lin et al., 2018; Cilz et al., 2019) to the human brain.

One unique aspect of our study is that we assessed an understudied feature of one’s social environment: interpersonal dynamics across the entire family. Prior studies have focused on the relation between overall hippocampal volume and the parent–child relationship with mixed results. One study found that maternal support during early childhood related to larger hippocampal volume measured at school age (Luby et al., 2012). Others have found a negative (Rao et al., 2010) or non-existent relationship (Whittle et al., 2014) between maternal support and hippocampal structure. These contradictory findings may be because measures of parental care do not sufficiently account for an individual’s family environment (Wang et al., 2017). While parental care undoubtedly affects how family members relate to one another, it is only one aspect of family function. Family systems theorists emphasize that examining a single parent–child dyad in isolation prevents a full understanding of the impact of social interaction patterns across the family (Cox and Paley, 1997; Lindsey and Caldera, 2006), and when entire-family interactions are quantified, they exhibit influences on children’s cognition and behavior (Gerstein and Crnic, 2018). Another possibility is that looking at potential associations with overall hippocampal volume may obfuscate relationships that exist at the level of individual hippocampal subfields. Research indicates hippocampal subfields are unique at both a cytoarchitectural (Insausti and Amaral, 2004) and functional level (Yassa and Stark, 2011; Schapiro et al., 2017; Schlichting et al., 2017). Given that some of these differences involve social hormones (Cilz et al., 2019) and social behaviors (e.g., Dudek et al., 2016), the structural sensitivity of the hippocampus to one’s social environment may be best examined at the subfield level. Our findings thus underscore that the dynamics of the entire family may play an important role in influencing specific aspects of the hippocampal circuit—namely the CA_1_ and CA_2/3_ subfields.

Our findings further suggest that family dynamics may exert an influence along a continuum, with both positive and negative family dynamics potentially influencing CA_1_ and CA_2/3_ structure. Negative interactions between parents and children have previously been shown to exert a long-term impact on cortisol responses, which are further associated with reduced hippocampal volume (Blankenship et al., 2019). A speculative interpretation of our findings might suggest that habitual, negative family dynamics—such as reduced family adaptability, increased family hardship, and negative communication skills—may elicit frequent stress responses, and lead to reductions in hippocampal volume. That interpersonal family dynamics related to CA_2/3_ volume is also noteworthy given evidence that CA_3_ particularly exhibits structural alterations elicited by chronic stress responses (Fuchs et al., 1995; McLaughlin et al., 2007).

At the opposite end of the continuum, positive family dynamics may protect or increase CA_1_ and CA_2/3_ volumes. Social bonding elicits social hormones that can alter synaptic transmission in the hippocampus (Cilz et al., 2019), and enriched environments can increase plasticity and neurogenesis within this region (Kempermann, 2008). Structural changes associated with synapse formation and neurogenesis may lead to increased hippocampal volumes, providing a speculative mechanism for the positive association between family dynamics and CA subfield volumes in the present study. A related body of work on the benefits of social support in humans complements the perspective that positive family dynamics may be neuroprotective. Robust social networks are associated with positive health outcomes (Berkman and Syme, 1979; Uchino, 2004), including resilience to stress (Cohen, 2004; Uchino, 2004; Ozbay et al., 2007). Collectively, these findings indicate that enriched social interactions in the context of the family may not only promote efficient function of the hippocampal circuit, but may also protect the circuit from the impacts of stress when it does arise, leading to larger hippocampal volumes. Notably, few studies in humans have directly measured how positive social environments impact hippocampal structure either overall or at the level of individual subfields (Rao et al., 2010; Luby et al., 2012; Whittle et al., 2014), instead focusing on the impacts of adverse social environments (Teicher and Samson, 2016; VanTieghem et al., 2021). Our data suggest that additional exploration of positive social interactions may be warranted to fully understand the mechanisms through which we can promote healthy brain development, improved memory function, and resilience to stress.

That family dynamics—a critical feature of our social world—showed a selective relation with CA_1_ and CA_2/3_ volumes highlights the particular sensitivity of these subfields to our social world. We predicted that CA_1_ and CA_2_ would be especially sensitive to social environment given their responsiveness to social hormones and stimuli (Okuyama et al., 2016; Rao et al., 2019). Our findings provide additional support for a role of these subfields in processing social experience, while also suggesting there may be long-term structural impacts on these regions that depend on one’s social environment. Though we cannot determine the directionality of the observed associations, it is intriguing to consider possibilities. We speculate that individuals with an enriched and positive social world may have enhanced plasticity in CA_1_ and CA_2_, as observed in rodents (Pagani et al., 2015; Lin et al., 2018), leading to long-term structural enhancements reflected in the increased CA_1_ and CA_2/3_ volumes observed here. However, it is also possible that individuals born with smaller CA_1_ and CA_2_ subfields may be predisposed to impairments in social cognition and thus have poorer social relations. While the role of genetics should also be considered, a solely genetic account seems unlikely. Prior work has established that hippocampal volume is a highly polygenic trait of only moderate heritability (Peper et al., 2007; van der Meer et al., 2020). Family dynamics are also synergistic, resulting from interactions across several individuals who may not even be biologically related (e.g., step-parents and step-siblings). Nonetheless, future work that incorporates both longitudinal and genetic approaches could provide important insight into the mechanisms underlying the relation between interpersonal family dynamics and both CA_1_ and CA_2/3_ volumes.

One notable aspect of our data is that family dynamics predicted CA_1_ and CA_2/3_ volumes across our entire sample, including our adult participants. We hypothesized initially that family dynamics may be more predictive of hippocampal subfield volumes in children relative to adults given that the hippocampus undergoes key developmental changes in middle childhood (DeMaster et al., 2014; Daugherty et al., 2016; Schlichting et al., 2017; Riggins et al., 2018; Humphreys et al., 2019). Early-life experiences in rodents exert lasting epigenetic effects on the hippocampus (Benito et al., 2018; Kempermann, 2019; Zocher et al., 2020). In humans, parental nurturance at age 4 years, but not age 8 years, related to hippocampal volume in adolescence (Rao et al., 2010). A longitudinal study also found that maternal support during early childhood exerted a stronger effect on hippocampal volume trajectories than maternal support during school age (Luby et al., 2016). It is possible that the hippocampus is more sensitive to family dynamics early in development, but that we failed to see an effect of age because we did not sample children younger than 7 years, or because family dynamics remain fairly constant from childhood through adulthood. The age range of our sample may also have prevented us from observing increased hippocampal sensitivity during later developmental periods such as adolescence (see Hueston et al., 2017 for a relevant review). A perhaps more interesting possibility is that hippocampal structure tracks fluctuations in family dynamics across the lifespan. This possibility aligns with work showing continued hippocampal plasticity into old age (Sheppard et al., 2019), as well as malleable family dynamics (Stratton et al., 2014). While we cannot confirm either possibility, that we found a relationship through adulthood highlights a potentially powerful and enduring role of interpersonal family dynamics on CA_1_ and CA_2/3_ structure.

### Limitations and Future Directions

While our findings demonstrate robust relationships between interpersonal family dynamics and CA_1_ and CA_2/3_ volumes, there are some limitations of our approach that should be considered. Notably, the present study takes a cross-sectional approach, which limits our ability to track both interpersonal family dynamics and hippocampal subfield volumes over time, including through adolescence. As mentioned above, interpersonal family dynamics may remain fairly constant or may change across development. Furthermore, the influence of family dynamics on hippocampal structure may exhibit non-linear relationships with age that can only be captured via longitudinal approaches. In other words, hippocampal subfield structure may be more sensitive to interpersonal family dynamics at critical periods during an individual’s social development. Future studies quantifying the trajectories of social development, interpersonal family dynamics, and hippocampal subfield structure would help address this possibility.

Future studies may also benefit from collecting both self-report and observational measures of family function, as well as information about whom participants consider family when rating their interpersonal dynamics. Consistent with standard SCORE-15 administration instructions (Stratton et al., 2010; Jewell et al., 2013), we instructed participants ages 12 years and older to choose whom they wanted to count as their family when completing the questionnaire. These instructions shift away from the biological definition of family. However, some individuals may have still adhered to the heteronormative definition of this term, placing constraints on the social group they chose to evaluate (see Teh et al., 2017). Collecting detailed information about who individuals consider family when rating their family dynamics could therefore deepen our understanding of the present study findings.

Finally, the nature of our data limit our ability to assess how interpersonal family dynamics relate to memory and/or hippocampal subfield *function*. While each component study assessed memory function using distinct tasks, we did not have a consistent measure of memory across the studies that could be incorporated into our analyses. Yet, our work does suggest potential avenues for exploring how interpersonal family dynamics influence hippocampal-mediated behaviors. For instance, our data suggest that tasks that are particularly reliant on CA_1_ and CA_2/3_ computations and representations may be most likely to show relationships with interpersonal family dynamics. Such tasks may include statistical learning (Schapiro et al., 2012; Schlichting et al., 2017), memory-based inference (Schlichting et al., 2014; Zeithamova et al., 2016), and memory-based discrimination (Bakker et al., 2008; Yassa and Stark, 2011), which have each been associated with human CA_1_ and CA_2/3_ function in particular (Schapiro et al., 2017).

## Conclusion

In summary, our results indicate that CA_1_ and CA_2/3_ volumes track normative variation in interpersonal family dynamics in a cross-sectional, developmental sample. Family represents the earliest and most prevailing social system for most individuals. How we interact within our family system is woven into the fabric of our daily lives and, based on our data, perhaps even the biological processes underlying neural structure. Critically, one’s perceived family dynamics are sensitive to therapeutic change (Carr, 2000). Interventions designed to improve these interactions may therefore have benefits not only for social function, but also for memory behaviors that rely on the hippocampus (Rubin et al., 2014). Taken together, our findings advance our understanding of hippocampal sensitivity to one’s environment, providing new evidence that CA_1_ and CA_2/3_ structure tracks even normative variation in one’s social world.

## Data Availability Statement

The dataset presented in this study can be found at https://osf.io/hk8mp/?view_only=bc76097b1815401dad6b96a11b70b35d.

## Ethics Statement

This study was reviewed and approved by the Institutional Review Board (IRB) at The University of Texas at Austin. Written informed consent to participate in this study, as well as for the publication of any images or data included in this article, was provided by the participants’ legal guardian/next of kin (if child) or participant (if adult).

## Author Contributions

CC, ARP, and EB-A designed the research and wrote the manuscript. CC, HER, NLV, MMM, and LLS performed the research. EB-A, CC, and HER analyzed the data. HER and NLV provided the manuscript feedback. All authors contributed to the article and approved the submitted version.

## Conflict of Interest

The authors declare that the research was conducted in the absence of any commercial or financial relationships that could be construed as a potential conflict of interest.

## Publisher’s Note

All claims expressed in this article are solely those of the authors and do not necessarily represent those of their affiliated organizations, or those of the publisher, the editors and the reviewers. Any product that may be evaluated in this article, or claim that may be made by its manufacturer, is not guaranteed or endorsed by the publisher.
