# Supplementary material for: Interpersonal Family Dynamics Relate to Hippocampal CA Subfield Structure
Source: Front Neurosci. 2022 Jun 17;16:872101. doi: 10.3389/fnins.2022.872101 (PMC9247275; doi:10.3389/fnins.2022.872101)
Supplement: Supplementary file 1 [file Data_Sheet_1.docx]

***Supplementary Materials***

**1. Supplementary Methods**

**1.1 Screening Participants**

Participants were screened for psychiatric symptoms in the clinical range and lower than average IQ. The Child Behavior Checklist (CBCL; Achenbach, 1991) assessed psychiatric problems in children; children with a total problems T-score in the clinical range were excluded. The Symptom Checklist 90-Revised (SCL-90-R; Derogatis, 1977) assessed psychiatric problems in adults; adults with a Global Severity Index score one standard deviation above the normative sample mean were excluded. The Wechsler Abbreviated Scale of Intelligence, Second Edition (Wechsler, 2011) assessed IQ in all participants; its vocabulary and matrix reasoning subtests were administered to yield a Full Scale IQ-2 Subtest score. Participants with a score more than two standard deviations below the normative sample mean were excluded. Performance on screening measures for the final sample is shown in **Table 1**.

**1.2 Excluded Participants**

Eighty-one participants were enrolled in the study, but were excluded prior to participating in the scanning phase because they were either lost to follow-up, voluntarily withdrew, or met exclusion criteria. These criteria included, but were not limited to: psychiatric symptoms in the clinical range, lower than average IQ, disclosure of a developmental or psychological disorder, MRI contraindication (e.g., metal in the body), and discomfort with a mock MRI environment.

An additional 56 participants participated in the scanning phase of the study, but were later excluded for the following reasons: incomplete or missing behavioral data (*n* = 30), issues with the quality of the scan session data that precluded further processing (*n* = 13), voluntary withdrawal (*n* = 5), no anatomical scans collected (*n* = 3), late failure to meet inclusion criteria (e.g., aging out of the study; *n* = 3), and researcher oversight (*n* = 2).

**1.3 Excluded or Missing Data**

Twenty-one participants included in the final sample (16 children and 5 adults) had more than one data wave (questionnaires plus structural MRI scans) available because they had completed two or more subsidiary studies within the lab. When this occurred, only the participant’s earliest complete data wave was used in analyses, such that their other data wave(s) were excluded. This resulted in a dataset that was balanced across participants.

**2. Supplementary Tables and Figures**

**Supplementary Table 1.**

*Models exploring the influence of unique interpersonal family dynamics dimensions.*

| Models | AIC |
| --- | --- |
| CA_1_ models |  |
| *Overall model*: CA_1_ volume ~ Age + SES + Interpersonal family dynamics | 2115.396 |
| *Dimension model 1*: CA_1_ volume ~ Age + SES + Strengths and adaptability | 2115.669 |
| *Dimension model 2*: CA_1_ volume ~ Age + SES + Overwhelmed by difficulties | 2116.858 |
| *Dimension model 3*: CA_1_ volume ~ Age + SES + Disrupted communication | 2117.231 |
|  |  |
| CA_2/3_ models |  |
| *Overall mode1*: CA_2/3_ volume ~ Age + SES + Interpersonal family dynamics | 1806.286 |
| *Dimension model 1*: CA_2/3_ volume ~ Age + SES + Strengths and adaptability | 1808.663 |
| *Dimension model 2*: CA_2/3_ volume ~ Age + SES + Overwhelmed by difficulties | 1807.009 |
| *Dimension model 3*: CA_2/3_ volume ~ Age + SES + Disrupted communication | 1806.419 |

**Supplementary Table 2.**

*Best-fitting model of interpersonal family dynamics (main effects model).*

| Fixed effects | Std. beta | 95% CI | *t*-value | |
| --- | --- | --- | --- | --- |
| *Intercept* | 0.76 | 0.72 - 0.79 | 40.11*** | |
| Age | -0.07 | -0.23 - 0.10 | -0.80 | |
| Sex |  |  |  | |
| Male (reference) |  |  |  | |
| Female | 0.08 | -0.25 - 0.41 | 0.48 | |
| R2 | 0.01 | | | |
| Adj. R2 | -0.01 | | | |
| *Note.* ****p*<.001. | | | |  |

**Supplementary Table 3.**

*Best-fitting model of hippocampal subfield volumes (subfield interaction model).*

| Fixed effects | Std. beta | 95% CI | *t*-value |
| --- | --- | --- | --- |
| *Intercept* | 543.83 | 508.00 - 579.66 | 29.80*** |
| Age | 0.02 | -0.01 - 0.05 | 1.43 |
| SES | 0.00 | -0.03 - 0.03 | 0.22 |
| FamDyn | 0.00 | -0.04 - 0.05 | 0.17 |
| SubID |  |  |  |
| Posterior (reference) |  |  |  |
| CA_1_ | 2.41 | 2.37 - 2.45 | 110.07*** |
| CA_2/3_ | 0.15 | 0.11 - 0.19 | 6.89*** |
| DG | 1.31 | 1.26 - 1.35 | 59.71*** |
| Subiculum | 2.01 | 1.97 - 2.05 | 91.70*** |
| FamDyn * SubID |  |  |  |
| FamDyn * Posterior (reference) |  |  |  |
| FamDyn * CA_1_ | 0.06 | 0.01 - 0.10 | 2.58** |
| FamDyn * CA_2/3_ | 0.01 | -0.03 - 0.06 | 0.66 |
| FamDyn * DG | 0.00 | -0.04 - 0.05 | 0.13 |
| FamDyn * Subiculum | 0.00 | -0.04 - 0.04 | 0.05 |
| Conditional R2 | 0.96 | | |
| Marginal R2 | 0.94 | | |

*Note.* Abbreviations: Family Dynamics (FamDyn); Subfield ID (SubID). ****p*<.001. ***p*=.10.

**Supplemental References**

Achenbach, T. M. (1991). *Manual for the Child Behavior Checklist/4-18 and 1991*

*profile*. Burlington, VT: Department of Psychiatry, University of Vermont.

Derogatis, L. R. (1977). *The SCL-R-90 manual I: Scoring, administration and*

*procedures for the SCL-90*. Baltimore, MD: Clinical Psychometric Research.

Wechsler, D. (2011). *Wechsler Abbreviated Scale of Intelligence–Second Edition*

*(WASI-II)*. San Antonio, TX: NCS Pearson.
